# Triptorelin in the Relief of Lower Urinary Tract Symptoms in Advanced Prostate Cancer Patients: The RESULT Study

**DOI:** 10.1155/2015/978194

**Published:** 2015-01-28

**Authors:** Alexandre Peltier, Fouad Aoun, Vincent De Ruyter, Patrick Cabri, Roland Van Velthoven

**Affiliations:** ^1^Department of Urology, Jules Bordet Institute, 1000 Brussels, Belgium; ^2^Ipsen NV, Guldensporenpark 87, 9820 Merelbeke, Belgium

## Abstract

This prospective, noninterventional, open-label, multicentre, Belgian study assessed the prevalence of moderate to severe lower urinary tract symptoms (LUTS) in patients with locally advanced or metastatic prostate cancer scheduled to receive triptorelin therapy and its effects on LUTS were evaluated focusing on symptom relief and changes in quality of life (QOL) related to urinary symptoms (November 2006 to May 2010). Inclusion criteria were age >18 years, histologically confirmed advanced or metastatic prostate cancer, and life expectancy ≥12 months. Exclusion criteria were treatment with any LHRH analogue within the last 6 months or any other investigational agent within the last 3 months before study entry. Patients who received one or more triptorelin doses and had one or more efficacy assessments were evaluated. In total, 325 patients were included with a median age of 74 years (50 to 95 years). Mean age at first diagnosis was 73 ± 8 years. Moderate (IPSS 8–19) to severe (IPSS ≥ 20) LUTS were observed in 62% of patients. Triptorelin reduced LUTS severity. This improvement was perceived within the first 24 weeks of treatment and was maintained after 48 weeks. A decrease in PSA level was also observed.

## 1. Introduction

Androgen deprivation therapy (ADT) for prostate cancer is 75 years old and its use has markedly increased in the last two decades in Western countries. In the United States, this treatment is administered to approximately 600,000 prostate cancer patients [1]. Similarly, in Australia, the use of ADT has increased by more than 40% from 2003 to 2009 [2]. ADT has been the basis for two Nobel prizes, the first to Charles Huggins for his seminal work and the second to Andrew Schally for the discovery of the luteinizing hormone releasing hormone (LHRH) agonists. Subsequently, LHRH agonists have become widely accepted as first line therapy for symptomatic metastatic prostate cancer or in combination with radiotherapy for locally advanced prostate cancer [3, 4].

Benign prostatic hyperplasia (BPH) is the main cause of lower urinary tract symptoms (LUTS) in ageing men. BPH and prostate cancer are common conditions in older men and there are similarities between the diseases [5]. The prevalence of both BPH and prostate cancer increases with age [6]; both are androgen dependent and both respond to ADT [7, 8]. In fact, men with bothersome LUTS and/or increased prostatic volume are more likely to be diagnosed with prostate cancer [9]. The increased diagnostic intensity between BPH/LUTS and prostate cancer is in part due to urological society guidelines, which recommend both digital rectal examination (DRE) and PSA testing for all patients with >10 year life expectancy in the basic evaluation of LUTS [10, 11]. Additionally, men with symptomatic BPH/LUTS who receive PSA assessment are likely to have an elevated PSA due to an enlarged prostate, and ultimately, these men are more likely to undergo subsequent biopsy [12]. Furthermore, there is compelling evidence from experimental and clinical studies that LHRH agonists can reduce total prostatic volume and improve voiding in patients with prostate cancer [12]. However, there is limited information from clinical trials on the prevalence of bothersome LUTS in patients with prostate cancer in day to day practice and only limited data are available on the impact of LHRH agonists on LUTS. Therefore, the objective of this noninterventional, multicentre, prospective, open-label study was to assess the prevalence of bothersome LUTS in patients with prostate cancer scheduled to receive ADT and to study the effects of this treatment on LUTS relief and changes in quality of life (QOL) related to improvements in urinary symptoms as the primary endpoint.

## 2. Materials and Methods

The present trial was a prospective, noninterventional, multicenter, open-label study performed in 26 centres in Belgium between 27 November 2006 and 11 May 2010 (trial identifier I-48-52014-150). The inclusion criteria were men aged > 18 years, with histologically confirmed prostate cancer (any stage) who were scheduled to receive an LHRH agonist (triptorelin 3.75 mg and/or 11.25 mg) within one month and with a life expectancy of at least 12 months as assessed by the treating physician, using a risk estimation tool of his or her preference (including clinical expertise, nomograms, epidemiological data, guidelines or other). All patients were treated with an oral antiandrogen 2 weeks before the instauration of the LHRH agonist in order to prevent flare-up; this antiandrogen treatment was stopped 2 to 4 weeks later. Patients who were treated with any LHRH analogue therapy and/or 5 alpha-reductase inhibitor and/or an investigational medicinal product within the last 3 months before study entry were excluded. Patients had been treated with triptorelin for a minimum of 1 year (4 injections of 11.25 mg, one every 12 weeks, or 12 injections of 3.75 mg, one every 4 weeks; patients were free to switch treatment schedule upon doctor's advice). Patients were asked to provide a signed written informed consent and inclusion and exclusion criteria were checked prior to study enrolment. The trial was carried out in compliance with the Helsinki declaration and good clinical practice. The study was approved by the leading Ethics Committee of the Bordet Institute in Brussels (IRB b40320072448).

Patient characteristics in terms of age, Gleason score, and TNM staging were gathered before therapy (Table 1). LUTS was assessed by the International Prostate Symptoms Score (IPSS) before initiation of the therapy and 24 and 48 weeks after the start of the treatment. Mild LUTS was defined as IPSS <7, moderate LUTS as IPSS between 8 and 19, and severe LUTS as IPSS ≥20. A clinically meaningful response was defined as an IPPS change from baseline of >3 points [13]. QOL was assessed through the separate last question of the IPSS-form (Question 8: If you were to spend the rest of your life with your urinary condition the way it is now, how would you feel about that?). Each of the variables was evaluated for the past month for the defined time points (baseline, 24 and 48 weeks).

Descriptive qualitative and quantitative statistics were used for analysis of the variables. Changes from baseline were assessed using paired tests (i.e., McNemar and Bhapkar's tests). Specifically the distribution of total IPSS categories at baseline, week 24, week 48, and at the last available visit, and the changes from baseline was analysed using descriptive qualitative statistics. 95% CIs for proportions were provided. Changes were assessed using Bhapkar's test of marginal homogeneity. To assess the correlation between total IPSS and PSA, a Spearman's correlation coefficient was used.

**Figure 1:**
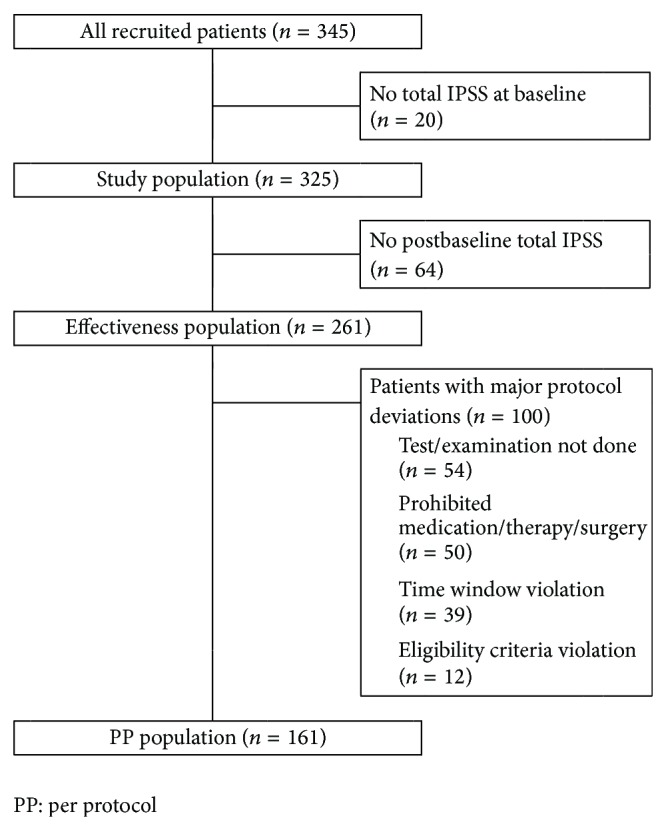
Patient disposition in the study.

All patients with a valid total IPSS measurement at baseline (*n* = 325) were included in the study (Figure 1). The effectiveness population included all patients who received at least one dose of triptorelin and had at least one postbaseline total IPSS efficacy assessment (*n* = 261). Patients from the effectiveness population without major protocol violations were included in the per protocol (PP) population (*n* = 161).

## 3. Results

### 3.1. Demographics

In total, 325 patients were included in this study with a median age of 74 years (range: 50 to 95 years) (Table 1). Mean age at first prostate cancer diagnosis was 73 ± 8 years. All but two patients were Caucasian. Triptorelin treatment was mainly indicated as first line therapy for locally advanced tumours (42%). Tumour stage was T3 for 75% of the patients, regional lymph node stage was N0 for 57%, and metastasis stage was M0 for 63%. At least one high risk characteristic was reported for 285 patients (89%): 11% of the patients had metastasis, 82% had a primary tumour stage of T3 or T4, 31% had a Gleason score ≥8, and 26% had a PSA result >20 ng/mL. Overall, 117 patients (36%) had previously been treated, mainly by radical prostatectomy (41%) or transurethral resection of the prostate (TURP; 39%), and some had previously received radiotherapy (24.8%).

At baseline visit, patients were mainly treated with triptorelin 11.25 mg combined with an antiandrogen therapy (46%) or with triptorelin 11.25 mg alone (35%). The most frequent concomitant treatments were nonsteroidal antiandrogens reported for 146 patients (45%), particularly bicalutamide (136 patients, 42%). Alpha-adrenoreceptor antagonists were reported for 51 patients (16%), particularly tamsulosin (31 patients, 10%), and steroidal antiandrogens were reported for 33 patients (10%), particularly cyproterone acetate (33 patients, 10%). At both weeks 24 and 48, most patients (81%) were treated with triptorelin 11.25 mg only. At the end of the study, 64 patients had incomplete data. In total, 261 patients with complete data made up the final cohort (Figure 1).

### 3.2. IPSS

At baseline, mean total IPSS score was 10.3 ± 6.4 (*n* = 325). More than half of the patients (169/325) had moderate symptoms and 31 patients out of 325 (9.5%) presented severe symptoms at baseline. In total, 200 patients (61.5%) presented moderate to severe LUTS at baseline. For 36 patients, IPSS was not assessed after baseline, leaving 164 patients for further evaluation (Table 2) with a mean total IPSS score of 14.0 ± 5.3 at baseline. The mean total IPSS score at week 24 decreased to 10.2 ± 4.6, corresponding to a change from baseline of −3.8 ± 4.8 points. At week 48, similar results were obtained with a mean total IPSS score of 9.8 ± 5.1 (change of −3.9 ± 6.2 points). Decreases in the obstructive IPSS subscore (−2.3 ± 3.3 points at week 24 compared with baseline) were primarily responsible for the decrease of the total IPSS score (Table 2).

### 3.3. Effect of Triptorelin on Total IPSS

**Figure 2:**
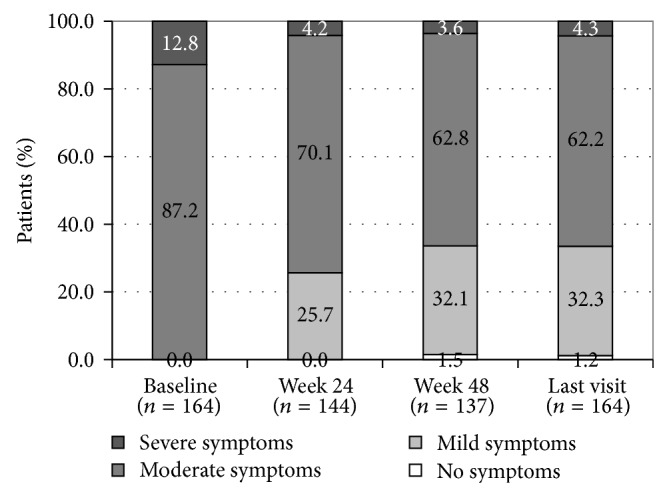
Proportions of triptorelin-treated patients with moderate to severe LUTS at baseline, week 24, week 48, and last visit.

Of the 164 patients with moderate to severe symptoms (63% of the effectiveness population), 143 (87.2%) had moderate symptoms, while 21 (12.8%) showed severe symptoms at baseline (Figure 2). At week 24, the distribution of patients according to the intensity of symptoms changed significantly (*P* < 0.001, Bhapkar's test). At this stage, 25.7% of these patients (*n* = 37/144) improved to no or mild symptoms. At baseline, these patients had either moderate (*n* = 34; 23.6%) or severe symptoms (*n* = 3; 2.1%) (Table 3). Additionally, 10 patients (6.9%) with severe symptoms at baseline had moderate symptoms at week 24. Only one patient (0.7%) with moderate symptoms at baseline worsened to severe symptoms at week 24. For the other patients, the intensity of symptoms was similar at baseline and week 24.

Also at week 48, the distribution of patients according to the intensity of symptoms changed significantly (*P* < 0.001; Bhapkar's test) compared to baseline. Among the patients with available data at week 48 (*n* = 137), 44 (32%) with moderate symptoms at baseline and 2 (2%) with severe symptoms at baseline had no or mild symptoms at week 48. For 11 patients (8%) with severe symptoms at baseline, the intensity of the symptoms had decreased to moderate symptoms at week 48. Three patients (2%) with moderate symptoms at baseline had severe symptoms at week 48. The intensity of symptoms was similar at baseline and week 48 for 75 patients (55%) with moderate symptoms and 2 patients (1%) with severe symptoms. Finally, the distribution of patients according to intensity of symptoms at the last available visit was similar to those described at week 48.

**Figure 3:**
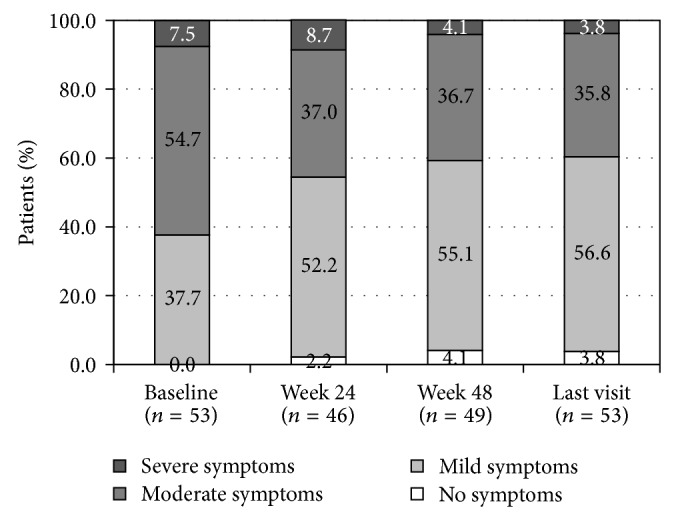
Proportions of triptorelin-treated patients having undergone radiotherapy or TURP, at baseline, week 24, week 48 and last visit.

In the subgroup of patients who had radiotherapy or TURP at baseline (*n* = 53), 60% had mild or no symptoms at week 48 (Figure 3). Also in this population, the distribution pattern according to the intensity of symptoms changed significantly at weeks 24 (*P* = 0.017) and 48 (*P* = 0.027) and at last visit (*P* = 0.005) compared to baseline (Table 4).

### 3.4. Effect of Triptorelin on Total PSA

The median PSA level of patients with moderate to severe LUTS (*n* = 164) decreased from 10.3 ng/mL (range: 0 to 4400 ng/mL) at baseline to 0.4 ng/mL (range: 0 to 215 ng/mL) at week 24, with a median change of −9.6 ng/mL. Similarly, at week 48, the median PSA was 0.1 ng/mL (range from 0 to 137 ng/mL, *n* = 143) with a median change from baseline of −9.2 ng/mL. At the last available visit, the median PSA was 0.2 ng/mL (*n* = 160), ranging from 0 to 137 ng/mL.

At baseline, 19 patients (*n* = 19/140; 13.6%) with moderate or severe LUTS had a PSA level <4 ng/mL (Table 5). The number of patients with PSA <4 ng/mL increased to 123 patients (*n* = 123/140; 87.8%) at week 24 and 130 patients (*n* = 130/142; 91.5%) at week 48. At the last available visit, 142 patients (*n* = 142/157; 90.4%) with moderate or severe LUTS had a PSA level <4 ng/mL. The changes in the distribution of patients according to PSA level are statistically significantly different (*P* < 0.001 Bhapkar's test) at all time points compared with baseline.

A weak correlation was observed between the change in total IPSS score from baseline to week 48 and change in PSA level from baseline to week 48 for patients with moderate to severe LUTS at baseline (*r* = 0.17; *P* = 0.047). In the overall effectiveness population, there was no correlation between change in total IPSS score and change in PSA level from baseline to each visit.

### 3.5. Effect of Triptorelin on QOL

At baseline, patients with moderate to severe LUTS had a mean score of 2.9 ± 1.1 at the last question of the IPSS score (QOL). At week 24, there was a mean decrease in this score from baseline of −0.8 ± 1.1 (*P* < 0.05). Similar decreases from baseline were reported at week 48 and at the last available visit (both time points: −0.9 ± 1.3; *P* < 0.05), showing an improvement in QOL related to urinary symptoms.

## 4. Discussion

The main objective of the present study was to estimate the prevalence of LUTS in patients with prostate cancer scheduled to receive triptorelin (3.75 mg and/or 11.25 mg) as part of standard ADT and to assess the effectiveness of triptorelin on relief of urinary symptoms and related QOL improvements over a 48-week treatment period. The prevalence of moderate to severe LUTS in patients with locally advanced or metastatic prostate cancer was 62% in this study. The primary endpoint was successfully met with a statistically significant LUTS relief (i.e., decrease of IPSS) and changes in QOL from baseline. The magnitude of the decrease was clinically meaningful with improvements of >3 points in the symptom score from baseline [13]. The rapid decrease in total IPSS, mostly attributable to improvements in voiding symptoms, could provide additional benefits for those complaining of obstructive LUTS at treatment initiation and could also facilitate the delivery of radiotherapy. This improvement was stable over time as shown by the statistically significant IPSS change from baseline at 48 weeks of treatment. Mean total IPSS improved from 14 ± 5 to 10 ± 5 at week 24 for patients with moderate to severe LUTS and from 10 ± 6 to 8 ± 5 for the overall effectiveness population, which also included patients with mild symptoms. Among the 164 patients with moderate (143 patients) to severe (21 patients) symptoms at baseline, 26% had mild symptoms at week 24 and 32% had mild symptoms or no symptoms (2%) at week 48. An improvement from severe to moderate symptoms was also observed for 8% of the patients. Most patients (55%) with moderate symptoms at baseline remained at this stage under treatment with triptorelin.

An improvement in QOL due to changes in urinary symptoms of patients with moderate to severe LUTS at baseline was also shown, as could be expected with an improvement in symptom intensity. The relief from symptoms was clearly associated with significant QOL improvements from baseline.

Generally, localised prostate cancer causes LUTS because most of the tumours arise in the periphery of the gland and progress toward the capsule more often than toward the urethra lumen [14]. LUTS could arise from locally advanced prostate cancer when the tumour invades the prostatic urethra, the bladder, or the neurovascular bundles [14, 15]. In day-to-day practice, patients with LUTS/BPH undergo an intensive diagnostic process that is responsible for the increased incidence of LUTS reported in patients with localised prostate cancer compared with the general male population. However, the prevalence of bothersome LUTS among patients with locally advanced and metastatic prostate cancer has not been commonly reported. In our study, the prevalence of patients with moderate to severe symptoms as assessed by total IPSS >7 was 62%. In a comparative study between goserelin and bicalutamide versus degarelix, Axcrona et al. reported similar results with 62.6% and 14.5% of their patients having moderate or severe LUTS, respectively [16].

Several studies have showed that surgical or biological castration improves voiding ability in patients with prostate cancer [14, 17, 18]. The improvement was fast occurring during the first month of therapy and stable with time even in patients with local progression [19].

If applied in patients with BPH, the effect of ADT might be explained by an overall reduction of the prostate volume. In patients with locally advanced prostate cancer, the effect could be related to tumour volume reduction rather than prostate volume reduction. In 1994, Mommsen and Petersen [17] showed that 62% (43/69) of patients with prostate cancer with acute urinary retention regained their voiding ability within 3 months after surgical castration. Even though patients treated with radiotherapy or TURP in our study had a significantly lower IPSS compared to the overall population, change in IPSS from baseline was statistically significant (*P* < 0.001). This could be explained by the tumour shrinkage effect or by an indirect action of triptorelin on the bladder. The statistically significant improvement of IPSS from baseline in patients with radical prostatectomy treated with triptorelin supports this hypothesis. However, this remains to be investigated* in vitro* and in large scale* in vivo* studies.

Effectiveness of treatment with triptorelin was also assessed by changes in PSA level. There was a large interindividual variability in PSA level, which has also been observed in many other studies [17–19], but a decrease in PSA level was observed for a large majority of the patients. While only 12% of the patients with moderate to severe LUTS had a PSA level <4 ng/mL at baseline, this increased to 88% at week 24 and 92% at week 48. There was a weak correlation between the change in total IPSS and the change in PSA level from baseline to week 48 for patients with moderate to severe LUTS at baseline (*r* = 0.17; *P* = 0.047), but there was no correlation between change in total IPSS score and change in PSA level from baseline to each visit in the effectiveness population overall.

This prospective, multicentre study examined urinary symptoms scores, PSA reductions, and outcomes. There are some limitations which should be taken into account when evaluating these results. Specifically the limitations related to the study type, including the lack of randomization and the absence of a control arm, and the inclusion of patients with LUTS who had undergone radical prostatectomy and/or TURP. Additionally, some data points were missing and IPSS has not been validated for LUTS attributable to causes other than BPH.

## 5. Conclusions

This study showed a 62% prevalence of moderate to severe LUTS among patients with locally advanced or metastatic prostate cancer planned to be treated with triptorelin. Treatment with triptorelin showed an effectiveness to reduce LUTS severity and to improve QOL in patients with prostate cancer. This improvement was perceived within the first 24 weeks of treatment and the effect was maintained after 48 weeks. The clinical benefit of triptorelin in terms of providing clinically meaningful relief of LUTS warrants further exploration in future urodynamic investigations. The improvement in IPSS in patients with locally advanced prostate cancer treated by triptorelin could be related to tumour volume and/or prostate volume reduction. The improvement of IPSS from baseline after receiving triptorelin in patients already treated with TURP and radical prostatectomy suggests another mechanism of action of LHRH agonist that should be investigated.

## Figures and Tables

**Table 1:** Baseline characteristics of the study population.

	Total (*N* = 325)
Indication to start triptorelin treatment at baseline	
Neoadjuvant before radical prostatectomy	5 (1.5%)
Neoadjuvant before radiotherapy or brachytherapy	62 (19.1%)
Adjuvant after radical prostatectomy	12 (3.7%)
Adjuvant after radiotherapy or brachytherapy	4 (1.2%)
Rising PSA after radical prostatectomy	29 (8.9%)
Rising PSA after radiotherapy or brachytherapy	23 (7.1%)
Locally advanced, first line therapy	135 (41.5%)
Locally advanced, after antiandrogen therapy	7 (2.2%)
Metastatic, first line therapy	43 (13.2%)
Other	17 (5.2%)
Missing data	0
Age at first prostate cancer diagnosis (years)	
Mean (SD)	72.86 (8.26)
TNM staging: T	
T1	**10 (3.1%)**
T1	1 (0.3%)
T1a	1 (0.3%)
T1b	3 (0.9%)
T1c	5 (1.6%)
T2	**47 (14.7%)**
T2	26 (8.1%)
T2a	7 (2.2%)
T2b	9 (2.8%)
T2c	5 (1.6%)
T3	**241 (75.1%)**
T3	182 (56.7%)
T3a	43 (13.4%)
T3b	16 (5.0%)
T4	**22 (6.9%)**
T*X*	**1 (0.3%)**
Missing data	4
TNM staging: N	
N0	**178 (57.1%)**
N1	**43 (13.8%)**
N*X*	**91 (29.2%)**
Missing data	13
TNM staging: M	
M0	**199 (63.4%)**
M1	**35 (11.1%)**
M1	28 (8.9%)
M1a	1 (0.3%)
M1b	6 (1.9%)
M*X*	**80 (25.5%)**
Missing data	11
Gleason score	
≤6	106 (35.5%)
7	99 (33.1%)
≥8	94 (31.4%)
Missing data	26

**Table 2:** Evolution of the IPSS (total, irritative subscore, and obstructive subscore) for patients with moderate to severe LUTS at baseline.

	Total IPSS	Irritative IPSS	Obstructive IPSS
At baseline (*n* = 164)			
Mean ± SD	14.0 ± 5.3	6.5 ± 2.7	7.5 ± 3.8
At week 24 (*n* = 144)			
Mean ± SD	10.2 ± 4.6	5.0 ± 2.4	5.2 ± 3.1
Change from baseline ± SD (*P* = NS)	−3.8 ± 4.8	−1.5 ± 2.4	−2.3 ± 3.3
At week 48 (*n* = 137)			
Mean ± SD	9.8 ± 5.1	4.8 ± 2.6	5.1 ± 3.3
Change from baseline ± SD (*P* = NS)	−3.9 ± 6.2	−1.6 ± 3.1	−2.3 ± 4.0
At last available visit (*n* = 164)			
Mean ± SD	10.0 ± 5.2	4.8 ± 2.5	5.1 ± 3.3
Change from baseline ± SD (*P* = NS)	−4.0 ± 6.1	−1.6 ± 3.0	−2.4 ± 3.9

**Table 3:** Change in intensity of symptoms from baseline to each visit for patients from the effectiveness population with moderate to severe LUTS at baseline.

	At baseline (*n* = 164)	
	Moderate symptoms	Severe symptoms
At week 24 (*n* = 144)		
(*P* < 0.001^*^ versus baseline)		
No symptoms	0 (0%)	0 (0%)
Mild symptoms	34 (23.6%)	3 (2.1%)
Moderate symptoms	91 (63.2%)	10 (6.9%)
Severe symptoms	1 (0.7%)	5 (3.5%)
At week 48 (*n* = 137)		
(*P* < 0.001^*^ versus baseline)		
No symptoms	1 (0.7%)	1 (0.7%)
Mild symptoms	43 (31.4%)	1 (0.7%)
Moderate symptoms	75 (54.7%)	11 (8.0%)
Severe symptoms	3 (2.2%)	2 (1.5%)
At last available visit (*n* = 164)		
(*P* < 0.001^*^ versus baseline)		
No symptoms	1 (0.6%)	1 (0.6%)
Mild symptoms	51 (31.1%)	2 (1.2%)
Moderate symptoms	88 (53.7%)	14 (8.5%)
Severe symptoms	3 (1.8%)	4 (2.4%)

^*^Bhapkar's test for homogeneity.

**Table 4:** Change in intensity of symptoms from baseline to each visit for patients from the effectiveness population who underwent radiotherapy or a TURP.

	At baseline (*n* = 53)		
	Mild symptoms	Moderate symptoms	Severe symptoms
At week 24 (*n* = 46)			
(*P* = 0.017^*^ versus baseline)			
No symptoms	1 (2.2%)	0 (0%)	0 (0%)
Mild symptoms	14 (30.4%)	9 (19.6%)	1 (2.2%)
Moderate symptoms	1 (2.2%)	16 (34.8%)	0 (0%)
Severe symptoms	1 (2.2%)	1 (2.2%)	2 (4.3%)
At week 48 (*n* = 49)			
(*P* = 0.027^*^ versus baseline)			
No symptoms	2 (4.1%)	0 (0%)	0 (0%)
Mild symptoms	15 (30.6%)	12 (24.5%)	0 (0%)
Moderate symptoms	3 (6.1%)	13 (26.5%)	2 (4.1%)
Severe symptoms	0 (0%)	1 (2.0%)	1 (2.0%)
At last available visit (*n* = 53)			
(*P* = 0.005^*^ versus baseline)			
No symptoms	2 (3.8%)	0 (0%)	0 (0%)
Mild symptoms	15 (28.3%)	14 (26.4%)	1 (1.9%)
Moderate symptoms	3 (5.7%)	14 (26.4%)	2 (3.8%)
Severe symptoms	0 (0%)	1 (1.9%)	1 (1.9%)

^*^Bhapkar's test for homogeneity. Note: percentages are based on the number of patients with available responses. To operate Bhapkar's test, a frequency equal to 0 was replaced by 0.001.

**Table 5:** Change in PSA levels from baseline to each visit for patients with moderate to severe LUTS at baseline.

	At baseline (*n* = 164)		
	0 to <4 ng/mL	≥4 to <10 ng/mL	≥10 ng/mL
At week 24 (*n* = 140)			
(*P* < 0.001 versus baseline)			
0 to <4 ng/mL	19 (13.6%)	46 (32.9%)	58 (41.4%)
≥4 to <10 ng/mL	0 (0%)	1 (0.7%)	5 (3.6%)
≥10 ng/mL	0 (0%)	0 (0%)	11 (7.9%)
At week 48 (*n* = 142)			
(*P* < 0.001 versus baseline)			
0 to <4 ng/mL	17 (12.0%)	51 (35.9%)	62 (43.7%)
≥4 to <10 ng/mL	1 (0.7%)	1 (0.7%)	2 (1.4%)
≥10 ng/mL	0 (0%)	0 (0%)	8 (5.6%)
At last available visit (*n* = 157)			
(*P* < 0.001 versus baseline)			
0 to <4 ng/mL	18 (11.5%)	54 (34.4%)	70 (44.6%)
≥4 to <10 ng/mL	1 (0.6%)	1 (0.6%)	3 (1.9%)
≥10 ng/mL	0 (0%)	0 (0%)	10 (6.4%)
